# Caring for Communities: Comparing Health Care System Patient Populations to Regional Populations

**DOI:** 10.1007/s11606-025-09867-y

**Published:** 2025-09-17

**Authors:** John P. Powers, Timothy S. Carey, Taylor W. Hargrove, Aubrey Limburg, Victoria Udalova, Amy Shaheen, Robert Bowers, Emily R. Pfaff, Barbara Entwisle

**Affiliations:** 1 North Carolina Translational and Clinical Sciences Institute, University of North Carolina at Chapel Hill, Chapel Hill, USA; 2 Carolina Population Center, University of North Carolina at Chapel Hill, Chapel Hill, USA; 3 U.S. Census Bureau, Suitland, USA; 4 UNC Health Alliance, Morrisville, USA

**Keywords:** integrated delivery of health care, population characteristics, population health, social determinants of health, racial groups

## Abstract

**Background:**

Recent years have seen an increase in the number and size of integrated health care delivery systems in the USA. The size and sophistication of these systems afford a greater focus on population health, leading to a fundamental question: How do the patients of these systems compare to the underlying regional populations that the systems serve?

**Objective:**

To demonstrate an approach to answering this question for a large public integrated delivery system, with a particular focus on neighborhood social determinants of health (SDOH).

**Design:**

We present a descriptive, graphical comparison of the neighborhood characteristics of UNC Health patients and the overall population of North Carolina (NC).

**Subjects:**

We leveraged electronic health record data from a 5-year period for patients at UNC Health, an integrated health care delivery system focused on serving the NC population. Estimates for the NC population were obtained from the American Community Survey (ACS).

**Main Measures:**

Measures included neighborhood SDOH indices for NC census tracts derived from ACS data as well as race and ethnicity.

**Key Results:**

Overall, patients were more concentrated in neighborhoods with the least and greatest disadvantage. However, the density patterns of specific racial and ethnic groups across neighborhood SDOH scores were similar between the patients and NC population.

**Conclusions:**

Using a large, public integrated health care delivery system, we illustrate an approach for comparing the demographic and neighborhood characteristics of the patients of such a system and its underlying regional population using freely available data and open-source software. Our findings indicate many similar patterns between the health care system patients and regional population, but overall higher concentrations of patients in neighborhoods with the least and greatest disadvantage.

**Supplementary Information:**

The online version contains supplementary material available at 10.1007/s11606-025-09867-y.

## INTRODUCTION

The past 15 years have seen a rise in the number and size of integrated health care delivery systems in the USA.^1^ These systems are characterized by unified administration of hospitals as well as primary care and specialty practices, including integrated management, quality of care procedures, and electronic health records (EHR) systems.^2^ The electronic data warehouses of these systems have led to the ability to analyze large amounts of health care data to improve patient care and outcomes.

Providers, advocates, insurers, and states are increasingly focusing on leveraging the size and sophistication of the health care data of these integrated systems to enhance population health.^3^ With the greater scale and coverage of these systems, they can be regarded as providing health care to large, regional populations.^4^ It then becomes essential to evaluate how the patients of these systems, i.e., the systems’ *patient populations*, compare to their broader regional populations. For example, are some groups of individuals overrepresented or underrepresented? Are the barriers to good health the same in the patient population and the regional population?

Such barriers can be conceptualized as social determinants of health (SDOH): “the conditions in which people are born, grow, learn, work, play, live, and age, and the wider set of structural factors shaping the conditions of daily life.”^5^ SDOH have been shown to have significant impacts on population health outcomes.^6–9^ The potential of SDOH research to accelerate advances in health equity has led many institutes and centers of the National Institutes of Health (NIH) to prioritize further investigation of these factors.^10^ While previous research on health disparities has focused largely on inequalities by race and ethnicity,^11^ structural inequities have long shaped and differentiated pathways to health among racial and ethnic groups in the USA. Indeed, recent findings have highlighted how SDOH may be differentially distributed among racial and ethnic groups.^5,6,12^ In particular, adverse SDOH have been reported with higher prevalence among several ethnoracial groups relative to non-Hispanic White and non-Hispanic Asian adults.^12^ Therefore, investigating SDOH in the context of race and ethnicity is important to obtaining a clearer understanding of health inequities.

The overall objective of this paper is to provide a simple, low-cost template for evaluating the similarities and differences in population characteristics between the patient population of an integrated delivery system and the underlying regional population. We focus especially on neighborhood SDOH and differences by race and ethnicity. Such methods can and should be used to guide how health systems and states allocate resources to address health inequities, acknowledging that comprehensive population health solutions will need to consider the upstream economic, social, cultural, and political factors shaping health outcomes and inequities.^13,14^ While many integrated health care delivery systems have implemented assessments of SDOH, including questions asked of individual patients during care, here we focus on neighborhood indices of SDOH. These indices are derived from publicly available data, and they can be linked to both patient address information and neighborhood population estimates to compare the neighborhood characteristics of the patient and regional populations. Thus, the use of neighborhood indices greatly simplifies this approach and ensures its accessibility to a wider range of health care systems in the USA.

The current effort utilizes an ongoing collaboration between the U.S. Census Bureau’s Enhancing Health Data (EHealth) Program and the University of North Carolina at Chapel Hill (UNC-CH). In this paper, distributions of race, ethnicity, and their relationships to neighborhood SDOH are compared between patients of the UNC Health system and the overall North Carolina (NC) population. UNC Health is composed of a large academic health center in Chapel Hill, 18 hospitals, 8446 physicians, and 1054 clinical sites in 52 counties. As a state-owned system, it focuses on NC, with a mission to improve health and health care for all Carolinians. With approximately 2 million patients seen over any 2-year period, UNC Health cares for around 20% of the NC population, and 99% of patients have an in-state address.

## METHODS

This study is approved by the UNC-CH Institutional Review Board. See the Appendix for guidance and code for replicating these methods.

### Data

We combined data from two sources for this work: EHR data from UNC Health and estimates from the American Community Survey (ACS).^15^

A comprehensive UNC Health patient population was defined from the EHR data for a study period of 2018–2022. This dataset included patients meeting all of the following criteria: had a NC address in the study period, had at least two recorded encounters in the study period at least 30 days apart (as an indication of active use of the health care system), had a non-null birth date, and was not incarcerated (incarcerated n = 8,812 in the study period). This dataset (N = 2,119,733) represents people under care at UNC Health during this period. Race, ethnicity, and census tract corresponding with the most recent patient address from within the study period were extracted for each patient. Address information was not available for 706 patients (0.03%); thus, these patients were not included in analyses.

Regional population estimates for each of the 2,195 NC census tracts, stratified by race and ethnicity, were obtained from publicly available 2015–2019 ACS 5-year estimates. Census tracts are statistical subdivisions of counties that typically range in population from 1200–8000 developed to approximate neighborhoods.^16^ ACS data provide estimates of population counts based on a sample of the nation’s population. The Census Bureau collects ACS data from roughly 1 to 2% of the US population each year. Survey weights are applied to ensure that ACS estimates represent the entire population.

We also computed or obtained several SDOH indices at the tract level based on ACS 1-year and 5-year estimates from 2013–2019. These included the Area Deprivation Index (ADI),^17,18^ Social Vulnerability Index,^19^ Community Resilience Estimates,^20^ Neighborhood Concentrated Disadvantage Index,^21^ Neighborhood Deprivation Index,^22^ Social Deprivation Index,^23^ and percentage of households below the poverty level. A report commissioned by the Office of the Assistant Secretary for Planning and Evaluation was referenced to select these indices, which were available or could be computed from publicly available data at the level of tracts.^24^ Details on computing and obtaining these indices are available in the Appendix. Specific interpretations vary by index, but generally, these measures aim to represent neighborhoods’ socioeconomic disadvantage, deprivation, or vulnerability to disasters. Census tracts were used to link patients and NC population estimates to SDOH index scores.

**Figure 1:**
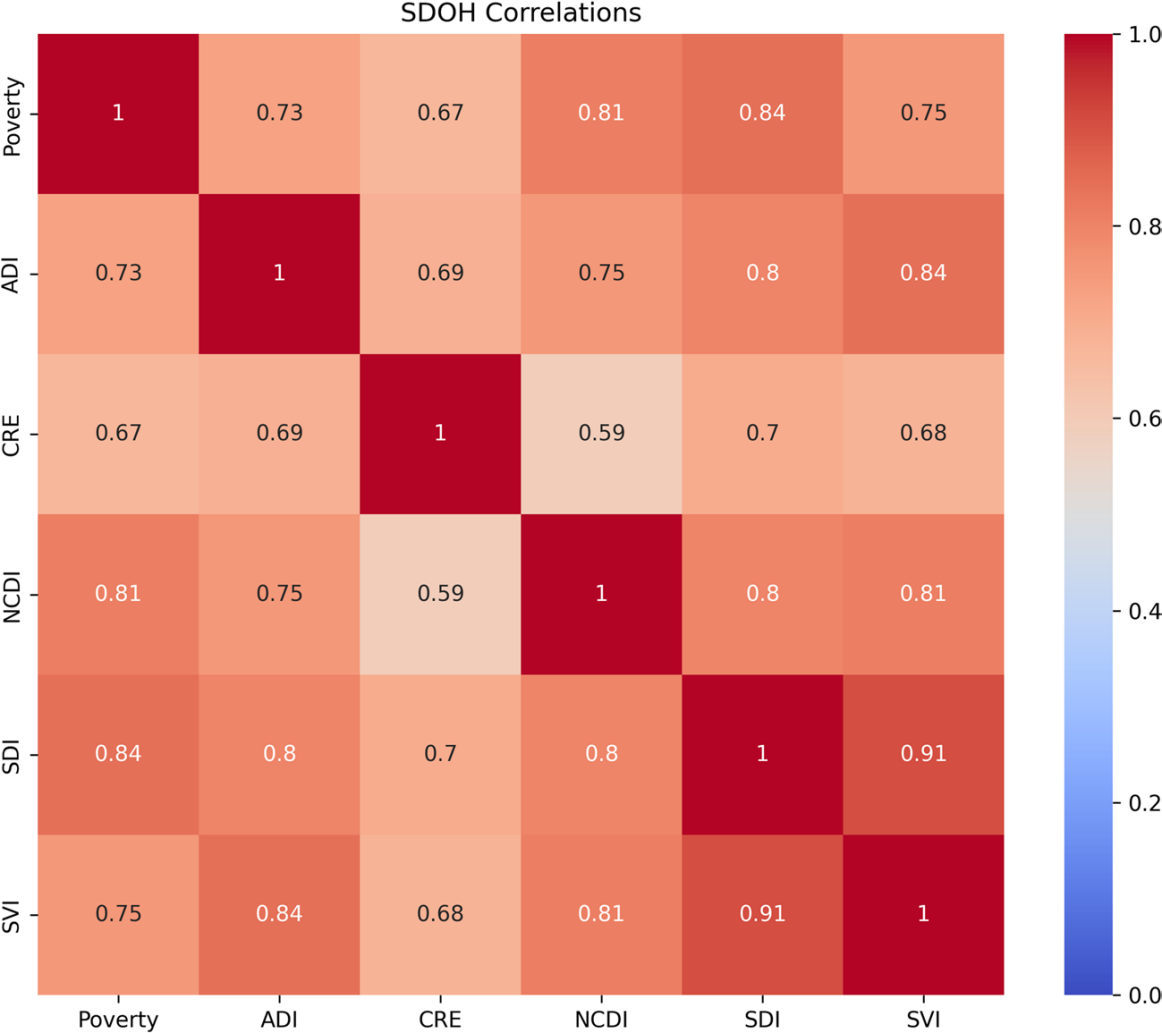
**Correlation matrix of SDOH indices in the patient population. Each Pearson correlation is based on all patients with values for both indices. Neighborhood Deprivation Index is not included as it is expressed as an ordinal measure with only five possible values. ADI, Area Deprivation Index; CRE, Community Resilience Estimates for 3 + risk factors; NCDI, Neighborhood Concentration Disadvantage Index; Poverty, percentage of households below the poverty level; SDI, Social Deprivation Index; SVI, Social Vulnerability Index.**

These various neighborhood SDOH indices were highly correlated with each other in the patient population, with most correlations (11 of 15) greater than or equal to 0.7 (see Fig. 1). Therefore, we focus in the main text on the results of a single index, the ADI. The ADI is an index of neighborhood socioeconomic disadvantage created to measure the impact of neighborhood characteristics on health disparities.^17^ The ADI has become a leading tool in health equity research and policy,^25,26^ and it does not involve any race-based measures in its computation, making it particularly suitable for race-related comparisons.

While the coding of ethnicity was the same between the EHR data and ACS estimates, there were some differences between these data sources in the coding of race. The EHR data for this project were provisioned from a prepared research dataset in the Observational Medical Outcomes Partnership (OMOP) common data model,^27^ which itself was derived from a prepared research dataset in the PCORnet common data model.^28^ As a result of these common data model transformations, the EHR dataset only included the following race categories: American Indian or Alaska Native (AIAN), Asian, Black or African American, Native Hawaiian or Other Pacific Islander, and White. Therefore, patients with a record of any other race category were missing a race value in this dataset, and patients with multiple races recorded had a race value in this dataset corresponding to only the race category listed first in their electronic health record. ACS estimates included the same five race categories as well as categories for *some other race alone* and *two or more races* (3.1% and 2.7% of NC population, respectively). These additional categories were excluded from analyses of race; to facilitate comparison, only categories present in both datasets were analyzed. For a detailed analysis of concordance between race and ethnicity data from UNC Health EHR versus the ACS, including categories of missing and other, see Rosa-Lebron et al., 2025.^29^

### Descriptive Analysis

Distributions of race and ethnicity for the patient and NC populations were summarized and compared. The distribution of patients across NC tracts was examined by plotting histograms of patient count per tract and patient count as a percentage of each tract’s population.

A set of kernel density estimation plots was generated for each SDOH index (or bar plots for Neighborhood Deprivation Index, an ordinal measure) using the Python package *seaborn* (v. 0.12.2).^30,31^ Kernel density estimation was used to provide visual representations of data distributions similar to histograms, except these representations use a smooth function rather than discrete bins, which facilitates visual comparison of several distributions in a single plot. For each index, one pair of plots was generated for the patient population and another for the NC population. In each pair, one plot illustrates the distribution of the population across tract index scores stratified by race and the other by ethnicity. This graphical approach to comparison was selected to illustrate similarities and differences across the full range of tract index scores and to circumvent problems with frequentist statistics related to extremely varied and often very large group sizes. All available data were used for each plot; i.e., only patients missing race data were excluded from plots based on race and likewise for ethnicity. Our main comparison of interest was between the patient and NC populations. Therefore, we also generated a kernel density estimation plot in which unstratified distributions for the patient and NC populations could be directly compared.

## RESULTS

Comparing the *Patients Excluding Missing* and *North Carolina Excluding Other* columns in Table 1, we observed similar overall distributions of race and ethnicity in the patient and NC populations.

**Table 1:** **Summary of Race and Ethnicity by Population**

	**Patients** **Count (%)** **2018–2022**	**Patients Excluding Missing** **Count (%)** **2018–2022**	**North Carolina** **Count (%)** **2015–2019**	**North Carolina Excluding Other** **Count (%)** 2015–2019
**Race**				
Total	2,119,733	1,815,425	10,264,876	9,674,837
American Indian or Alaska Native	40,491 (1.9%)	40,491 (2.2%)	123,952 (1.2%)	123,952 (1.3%)
Asian	44,109 (2.1%)	44,109 (2.4%)	292,992 (2.9%)	292,992 (3.0%)
Black or African American	431,074 (20.3%)	431,074 (23.7%)	2,200,761 (21.4%)	2,200,761 (22.7%)
Native Hawaiian or Other Pacific Islander	2,418 (0.1%)	2,418 (0.1%)	7,213 (0.1%)	7,213 (0.1%)
White	1,297,333 (61.2%)	1,297,333 (71.5%)	7,049,919 (68.7%)	7,049,919 (72.9%)
Missing/Other	304,308 (14.4%)	–	590,039 (5.7%)	–
**Ethnicity**				
Total	2,119,733	1,981,962	10,264,876	10,264,876
Hispanic or Latino	162,500 (7.7%)	162,500 (8.2%)	962,665 (9.4%)	962,665 (9.4%)
Not Hispanic or Latino	1,819,462 (85.8%)	1,819,462 (91.8%)	9,302,211 (90.6%)	9,302,211 (90.6%)
Missing	137,771 (6.5%)	–	–	–

The North Carolina columns represent population estimates based on American Community Survey samples rather than raw counts. The *Patients Excluding Missing* and *North Carolina Excluding Other* columns reflect the data included in analyses; the full *Patients* and *North Carolina* columns are included for completeness. The *Missing/Other* row indicates missing values for the patient dataset and *some other race alone* and *two or more races* values for the North Carolina estimates.

**Figure 2:**
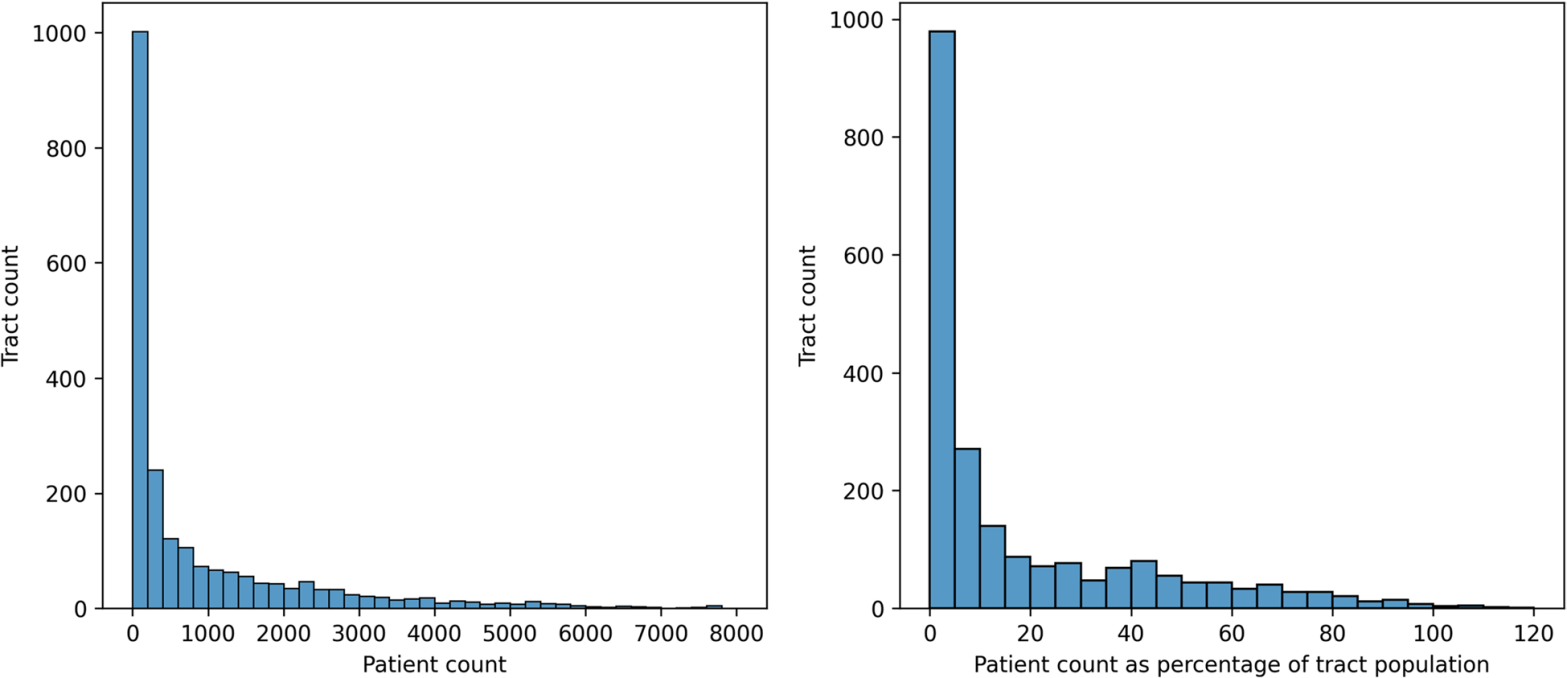
**Histograms of patient count by tract and patient count as a percentage of tract population. For the left panel, bin size is 200 patients. The horizontal axis is limited to 8000 patients. Seven tracts had patient counts above 8000 and are not shown on the plot. For the right panel, bin size is 5%. The horizontal axis is limited to 120%. Eleven tracts had percentages above 120% and are not shown on the plot. Patient count as a percentage of tract population can exceed 100% for various reasons. Tract populations are estimates based on 2015–2019 American Community Survey 5-year data. Patient counts are based on patients’ most recent address information on record during the study period of 2018–2022. Thus, percentages are derived from numerator and denominator estimates from different data sources and time periods that do not fully overlap. The earlier American Community Survey time window was required to use a common census tract map across SDOH indices.**

Regarding the distribution of the patient population across NC tracts, Fig. 2 illustrates substantial variability in the density of patients across tracts. A small number of tracts were densely populated with patients. For nearly half of the tracts though, fewer than 200 patients resided in the tract, and patients accounted for less than 5% of the tract’s population. Nevertheless, patients were present in 99% of NC tracts.

**Figure 3:**
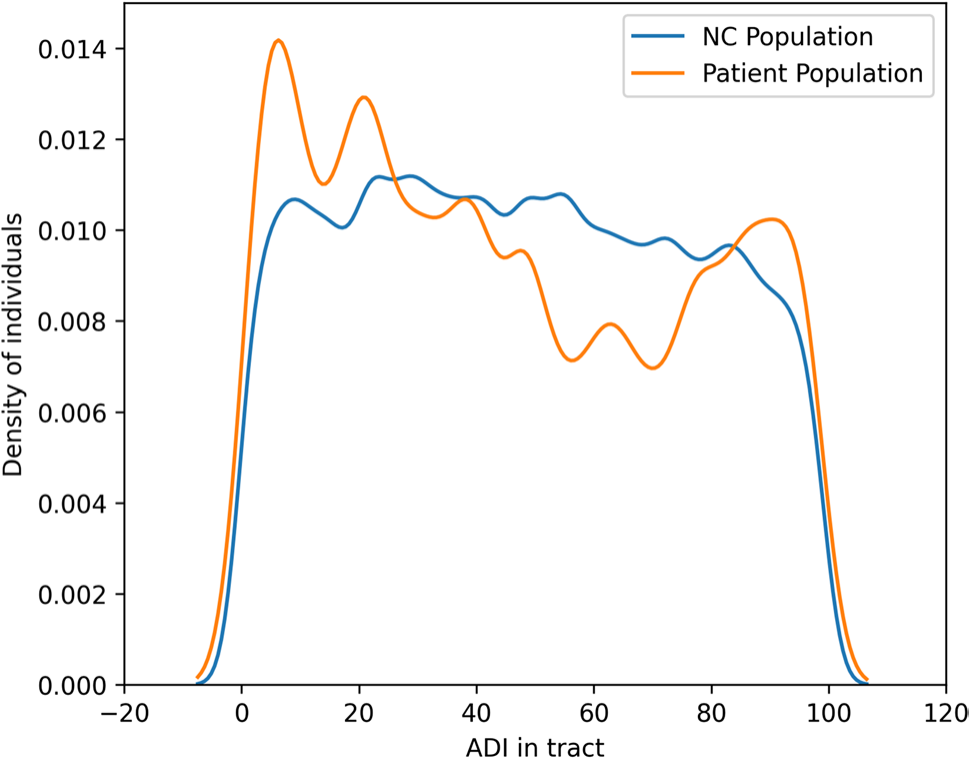
**Overall kernel density estimation plot for Area Deprivation Index. This plot shows how the concentration of patients or residents varies by the Area Deprivation Index score of the tract they live in. The Area Deprivation Index is expressed as percentile scores, with higher scores indicating greater deprivation. Thus, the horizontal axis indicates increasing tract deprivation going from left to right. Curves indicate the density of the populations across tract scores. Curves are individually normalized such that the area under each curve equals 1, allowing for relative density comparisons. ADI, Area Deprivation Index.**

**Figure 4:**
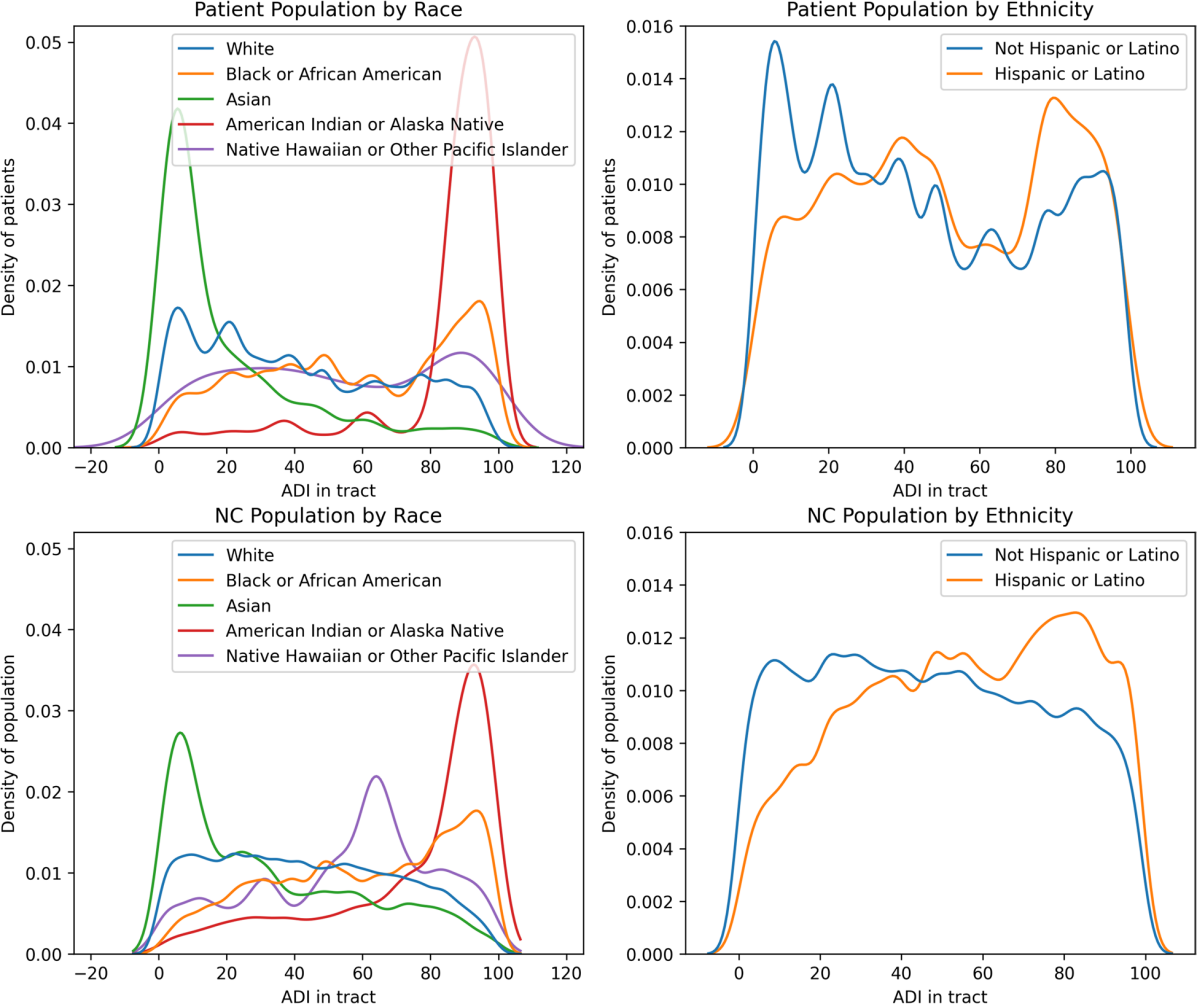
**Stratified kernel density estimation plots for Area Deprivation Index. These plots show how the concentration of patients or residents, stratified by racial or ethnic group, varies by the Area Deprivation Index score of the tract they live in. The Area Deprivation Index is expressed as percentile scores, with higher scores indicating greater deprivation. Thus, the horizontal axis indicates increasing tract deprivation going from left to right. Curves indicate the density of the groups across tracts scores. Curves are individually normalized by group size such that the area under each curve equals 1, allowing for relative density comparisons. ADI = Area Deprivation Index.**

The overall comparison for ADI indicated greater representation of the patient population relative to the NC population in the least and most deprived tracts, with 56.1% of patients residing in tracts in the top and bottom quartiles of ADI scores versus 47.6% of the NC population (see Fig. 3 and Table S1). Nevertheless, we observed similar patterns between the patient and NC populations for the SDOH distributions stratified by race and ethnicity (see Fig. 4 and Table S2). Figures for the other SDOH indices are included in the Appendix (Figures S1-6).

Racial and ethnic groups displayed distinct SDOH distributions (see Fig. 4 and Table S2), including a striking pattern of high deprivation for AIAN groups, especially in the patient population (82.8% in the top quartile of ADI scores). Asian groups were particularly concentrated in lower deprivation tracts (69.9% of patients in the bottom quartile of ADI scores). There were more subtle trends for greater deprivation in Black or African American groups and lower deprivation in White groups. Hispanic or Latino groups also showed a trend toward greater concentration in higher deprivation tracts relative to Not Hispanic or Latino groups. These patterns are clearly visible in the results for ADI (Fig. 4 and Table S2) and are generally consistent across SDOH indices (see Figures S1-6).

## DISCUSSION

We observed many similarities between characterizations of UNC Health patients and the greater NC population, despite highly variable representation of patients across the NC tracts. Specifically, the patient and NC populations were similar in distributions of race and ethnicity as well as distributions of racial and ethnic group density across neighborhood SDOH scores. For the overall distributions of the populations by neighborhood SDOH, however, we observed greater concentrations of patients in the least and most deprived neighborhoods and lower concentrations of patients in moderately deprived neighborhoods relative to the NC population.

When considering issues of population health care, the demographics and social needs of people seeking care in integrated health care delivery systems as compared to the regional populations become increasingly pertinent. State health systems, such as UNC Health, will generally target high similarity between these populations, and we did observe substantial similarity, especially in patterns related to race and ethnicity. The relatively greater concentration of patients overall in neighborhoods with the least and greatest disadvantage could be the result of several factors, including the variable distribution of UNC Health facilities and services in areas of low and high disadvantage and the UNC Health Financial Assistance Program, which makes the system a safety net provider. We do not assert, though, that all integrated delivery systems’ patient populations need to mirror the regional populations exactly. Some organizations may have institutional missions to serve particular population subgroups.

The similarities we observed between the patient and NC populations included consistent patterns of differences in SDOH among racial and ethnic groups. For race, we especially found high concentrations of Asian groups in neighborhoods of low disadvantage and high concentrations of AIAN groups in neighborhoods of high disadvantage. NC is home to a number of American Indian tribes and the largest indigenous population east of the Mississippi river.^32^ A large portion of this population lives in Robeson County (mostly Lumbee Tribe), which had the lowest median household income of any NC county in the 2015–2019 ACS 5-year estimates. Poor social and health outcomes are consistently reported for this population.^33–35^ For ethnicity, we observed greater density of Hispanic and Latino groups in neighborhoods of higher disadvantage relative to Not Hispanic or Latino groups, as expected.^36^

The development of standard methods for these comparisons will assist in planning and coordination as health care systems grow and work collaboratively with the public health and social services communities to improve population health.^3,37^ Such collaborations are critical as integrated health care delivery systems should be aware of the characteristics and needs of the populations they serve, although alone they are not positioned to remediate SDOH and their systemic causes.^13,14^ The methods employed here are low-cost and generalizable to any large integrated delivery system in the USA. They use basic data available from a system’s own EHR, ACS estimates publicly available at no cost, and SDOH indices derived from those freely available ACS estimates, and most of the analytic work can be performed with free, open-source software (see our shared code, https://github.com/NCTraCSIDSci/caring_for_communities). This same approach can also be extended to evaluate more specific patient populations within a system, for example, comparing patients screened for colon cancer or patients that have received a given vaccine to the regional population. Such approaches can be used to design and monitor targeted efforts to reduce inequities in care provision.^38^

The precision of our results is limited by differences in the coding of race between the patient dataset and the ACS. Due to these differences, patients identifying as more than one race were rolled into comparisons with the NC population identifying as one race alone. This conflation of patients identifying with multiple races with patients identifying with one race alone introduces some uncertainty into our comparisons, although the 2015–2019 ACS 5-year estimates indicate that less than 3% of individuals in NC identify as more than one race. Moreover, any systematic differences in the coding of race between the datasets might be expected to obfuscate similarities; however, we still observed strong similarities between the populations with respect to race.

As shown in Table 1, some patients were also missing data for race or ethnicity. A portion of the missing race data corresponds to patients with a record of other race, as the patient dataset had no category for other race. Therefore, neither patients nor NC residents identifying as other race were included in our comparisons. Other patients missing race or ethnicity data were simply missing these data in the EHR, and the effect of these missing data on our results is unknown. From previous work, we know that non-Hispanic White patients tend to be overrepresented in these missing data and other groups underrepresented.^29^ Nevertheless, the overall rate of missing race data here (14%) is actually lower than the average rate of many health care systems (20%).^28^

We used a minimum of only two inpatient or outpatient visits, potentially over multiple years, in the definition of our patient population. The amount of care received will of course be highly influenced by patient symptoms and conditions. We were interested in examining all levels of utilization for this work. Future analyses will examine the relationships of SDOH and utilization with specific conditions. In addition, this work focuses on neighborhood measures of SDOH. Neighborhood measures can efficiently capture the social resources or systemic disadvantage of a particular geographic area, but they may not reflect the circumstances of a given individual in that area. This is a limitation common to ecological studies. Therefore, future work will incorporate assessments of SDOH at the individual level.

The current paper presents a template to aid integrated delivery systems in assessing their care for communities by comparing the social characteristics of their patients to the surrounding regional population. We particularly highlight methods for comparing SDOH, given the growing focus on using knowledge of these factors to advance population health solutions. Our findings support further streamlining this low-cost approach through more targeted selection of SDOH indices, which will enable research within and across systems and regions. The suitability of the ADI as a representative SDOH index should be replicated in other systems. Our findings also emphasize the significant differences in SDOH that can be observed across racial and ethnic groups.

## Supplementary Information

Below is the link to the electronic supplementary material.

Supplementary Material 1 (DOCX 1.41 MB)

## Data Availability

The electronic health records data used in the analyses presented in this paper rely on a limited data set, as defined and protected by the Health Insurance Portability and Accountability Act Privacy Rule 45 CFR 164.514 issued by the U.S. Department of Health and Human Services and cannot be made publicly available. The publicly available ACS estimates we used can be obtained in a variety of ways (see https://www.census.gov/programssurveys/acs/data.html). We obtained estimates from the relevant Detailed Tables accessed through the ACS Summary File (https://www.census.gov/programs-surveys/acs/data/summary-file.html).
