# Supplementary material for: Caring for Communities: Comparing Health Care System Patient Populations to Regional Populations
Source: J Gen Intern Med. 2025 Sep 17;41(2):330–7. doi: 10.1007/s11606-025-09867-y (PMC12894526; doi:10.1007/s11606-025-09867-y)
Supplement: Supplementary file 1 — Supplementary Material 1 (DOCX 1.41 MB) [file 11606_2025_9867_MOESM1_ESM.docx]

**Appendix for:**

**Caring for Communities: Comparing Health Care System Patient Populations to Regional Populations**

**Supplementary Methods for SDOH Indices**

For the 2010-2019 decade, there were 2,195 census tracts in NC. We used this system of census tracts for all analyses. In the 2015-2019 ACS 5-year estimates (the set of ACS estimates we used to compute SDOH indices), 25 of these tracts had an estimated population of zero. These 25 tracts were omitted from all analyses, as most SDOH indices could not be computed for these tracts. Five additional tracts were estimated to have zero households and other tracts had very small numbers of households. Given that some of the SDOH indices below are based on household data, variable numbers of tracts were missing scores (see counts below) depending on the availability of the ACS estimates relevant to each index. Additional methods for each SDOH index are summarized below, and the sources of all SDOH indices are additionally summarized in Table S3.

***Area Deprivation Index (ADI)***

The ADI is an index of neighborhood socioeconomic disadvantage. The ADI is computed as a weighted sum of 17 component measures based on ACS data, which is then transformed into percentile scores for a given region. We computed ADI for census tracts in NC. Tracts missing data for three or more component measures were excluded from analysis. For tracts missing data for 1-3 component measures, missing values were imputed using an iterative imputation technique (IterativeImputer from the scikit-learn Python library) similar to the MICE method (multivariate imputation by chained equations). We followed the methods of Singh, 2003^17^ and Kind et al., 2014^18^ for component measure computation, weighting, and percentile scoring with one exception: the low and high income ranges used to compute the income disparity component measure were adjusted for inflation (<$10,000 was adjusted to <$20,000 and ≥$50,000 was adjusted to ≥$100,000). Final ADI scores were available for 2,158 NC tracts.

***Social Vulnerability Index (SVI)***

The SVI is an index of vulnerability to impacts of disasters and other community stressors. It is based on 15 component measures derived from ACS data. Communities within a region are ranked. Rankings are then converted to a 0-1 score scale. We obtained 2018 SVI scores for NC tracts from the CDC website (<https://www.atsdr.cdc.gov/place-health/php/svi/index.html>). SVI scores were available for 2,162 NC tracts.

***Community Resilience Estimates (CRE)***

The CRE, produced by the U.S. Census Bureau, are indices constructed from individual and household ACS data to measure vulnerability of areas to disasters. CRE are expressed as the percentage of individuals in an area with a given number of risk factors. For this analysis, we focus exclusively on the percentage of individuals with 3 or more risk factors living in an area in 2019 for 2,170 NC census tracts. These estimates are publicly available from the U.S. Census Bureau (<https://www.census.gov/programs-surveys/community-resilience-estimates.html>).

***Neighborhood Concentrated Disadvantage Index (NCDI)***

The NCDI is an index of socioeconomic disadvantage. The original index is a weighted sum of six component measures derived from ACS data. We computed it based on five component measures (excluding percentage of population that is Black or African American alone) following the methods described by the PhenX Toolkit (<https://www.phenxtoolkit.org/protocols/view/211302>).^39^ Final NCDI scores were available for 2,165 NC tracts.

***Neighborhood Deprivation Index (NDI)***

The NDI is an index of socioeconomic disadvantage. It is computed as a weighted sum of 10 component measures derived from ACS data, which is then transformed into quintile scores based on national tract data. We obtained data for 2017 NDI scores and component measures for NC tracts from the National Cancer Institute GIS Portal for Cancer Research (<https://www.gis.cancer.gov/>). After censoring tracts missing values for many component measures due to having very small numbers of households, missing values for remaining tracts were imputed using an iterative imputation technique (IterativeImputer from the scikit-learn Python library) similar to the MICE method (multivariate imputation by chained equations). Final NDI scores were available for 2,158 NC tracts.

***Social Deprivation Index (SDI)***

The SDI is an index of social disadvantage. It is computed as a weighted sum of 7 component measures based on ACS data, which is then transformed into percentile scores. We obtained data for 2019 SDI scores for NC tracts from the Robert Graham Center website (<https://www.graham-center.org/maps-data-tools/social-deprivation-index.html>). SDI scores were available for 2,170 NC tracts.

***Poverty***

Using the ACS data, we computed the percentage of households below the poverty level for each tract. Scores were available for 2,165 NC tracts.

**Replication Guidance**

This method requires data from two sources: the EHR of the health care system of interest and ACS estimates.

EHR data will be obtained from the data warehouse corresponding to the EHR system. The EHR dataset will be constrained to a study period of interest. The EHR dataset must be filtered to patients with residential address data, as these location data are required to link patient EHR data to area-level ACS estimates. Patient addresses must be geocoded and converted to their corresponding geographic level of interest (e.g., tracts). At UNC-CH and UNC Health, the software Maptitude is used to perform this address geocoding and conversion to tracts. The researchers will define and implement inclusion and exclusion criteria to define the patient population of interest. Other than location data and any data elements needed for inclusion and exclusion criteria, the researchers can include any data elements of interest for characterizing the patient population (e.g., race, ethnicity) that will also be available through ACS estimates for characterizing the regional population.

Publicly available ACS estimates can be obtained in a variety of ways (see <https://www.census.gov/programs-surveys/acs/data.html>). We downloaded relevant Detailed Tables accessed through the ACS Summary File (<https://www.census.gov/programs-surveys/acs/data/summary-file.html>). Estimates will be obtained for a time period corresponding to the study period of the EHR dataset. Researchers should consider that tract boundaries are revised every decade. Thus, if the study period spans a decade break, the researchers must decide whether to use census tract estimates corresponding to the earlier or later decade. For example, our EHR study period was 2018-2022, so we used the 5-year Summary File for 2015-2019 since a new system of tracts went into effect starting in 2020. When selecting a study period, researchers should also note that ACS 5-year estimates include more coverage of lower-level geographies such as tracts relative to 1-year estimates (see <https://www.census.gov/programs-surveys/acs/guidance/estimates.html>). All required ACS estimates will then be filtered to geographies of interest (e.g., all tracts of a given state, or tracts in select counties).

In our case, we used ACS estimates to obtain both corresponding regional characteristics to those in the EHR data for comparison (e.g., race and ethnicity breakdowns and total population per tract) as well as areal SDOH indices. For basic characteristics, researchers will decide on an approach for resolving any differences in how the data are coded in the ACS versus the EHR data, as we did and describe in the main text. For SDOH indices, these are sometimes already computed and available for the appropriate period and geography from publicly available sources. Otherwise, many areal SDOH can be manually computed from the publicly available ACS estimates.

Areal data (in our case, areal SDOH indices) will then be linked to the regional characteristics and EHR patient data characteristics by geography. Depending on how the EHR dataset is defined, researchers will likely need to decide how to handle cases where patients who have moved during the study period are associated with multiple geographies. Finally, patient and regional characteristics will be tabulated, plotted, and compared. We provide the Python and PySpark code we used to implement the above methods at <https://github.com/NCTraCSIDSci/caring_for_communities>, focusing specifically on the ADI as the areal SDOH index of interest.

In terms of estimating effort and cost for the work described above, these will vary based on several circumstances. Based on our experience, we estimate that most of these steps could be implemented by a single data scientist or data analyst over a period of 1-3 months, especially by utilizing the code we provide. Variable factors include how efficient the health care system’s process is for preparing research datasets from their EHR data warehouse and any costs associated with this; whether the health care system already has a geocoding process for their EHR data; the availability of a computing system that is appropriately powered to process and analyze the size of datasets involved (e.g., we used a computing system designed to leverage Apache Spark) and any costs associated with this; and the familiarity of the researcher(s) with obtaining and using public ACS estimates.

**Supplementary Tables**

|  | **ADI Score Bin** | | | |
| --- | --- | --- | --- | --- |
|  | [0, 25) | [25, 50) | [50, 75) | [75, 100) |
| **NC Population** | 25.7% | 27.0% | 25.3% | 21.9% |
| **Patient Population** | 31.9% | 26.0% | 17.9% | 24.2% |

Table S1. Overall quartile percentages for Area Deprivation Index. These percentages correspond to Figure 3 and indicate the proportion of each distribution in each quartile of ADI scores. ADI = Area Deprivation Index

|  | **ADI Score Bin** | | | |
| --- | --- | --- | --- | --- |
|  | [0, 25) | [25, 50) | [50, 75) | [75, 100) |
| **NC Population** | | | | |
| American Indian or Alaska Native | 7.5% | 11.1% | 16.4% | 64.9% |
| Asian | 48.7% | 23.6% | 16.7% | 11.0% |
| Black or African American | 13.2% | 23.5% | 25.4% | 37.8% |
| Native Hawaiian or Other Pacific Islander | 16.3% | 17.9% | 43.1% | 22.7% |
| White | 29.6% | 28.7% | 25.5% | 16.1% |
| Hispanic or Latino | 16.5% | 25.8% | 27.7% | 30.0% |
| Not Hispanic or Latino | 26.7% | 27.1% | 25.1% | 21.1% |
| **Patient Population** | | | | |
| American Indian or Alaska Native | 4.7% | 5.9% | 6.6% | 82.8% |
| Asian | 69.9% | 17.4% | 6.8% | 5.9% |
| Black or African American | 17.6% | 25.3% | 19.3% | 37.7% |
| Native Hawaiian or Other Pacific Islander | 24.0% | 24.7% | 16.8% | 34.5% |
| White | 36.4% | 26.8% | 18.3% | 18.5% |
| Hispanic or Latino | 23.0% | 28.1% | 17.6% | 31.3% |
| Not Hispanic or Latino | 32.3% | 25.8% | 17.9% | 24.0% |

Table S2. Stratified quartile percentages for Area Deprivation Index. These percentages correspond to Figure 4 and indicate the proportion of each distribution in each quartile of ADI scores. ADI = Area Deprivation Index

| **SDOH Index** | **Source** | **Data Year(s)** |
| --- | --- | --- |
| Area Deprivation Index | Study team computed from ACS 5-year estimates | 2015-2019 |
| Social Vulnerability Index | CDC website, <https://www.atsdr.cdc.gov/place-health/php/svi/index.html> | 2018 |
| Community Resilience Estimates | U.S. Census Bureau website, <https://www.census.gov/programs-surveys/community-resilience-estimates.html> | 2019 |
| Neighborhood Concentrated Disadvantage Index | Study team computed from ACS 5-year estimates | 2015-2019 |
| Neighborhood Deprivation Index | National Cancer Institute GIS Portal for Cancer Research, <https://www.gis.cancer.gov/> | 2017 |
| Social Deprivation Index | Robert Graham Center website, <https://www.graham-center.org/maps-data-tools/social-deprivation-index.html> | 2019 |
| Poverty | Study team computed from ACS 5-year estimates | 2015-2019 |

Table S3. Summary of SDOH index sources.

**Supplementary SDOH Figures**


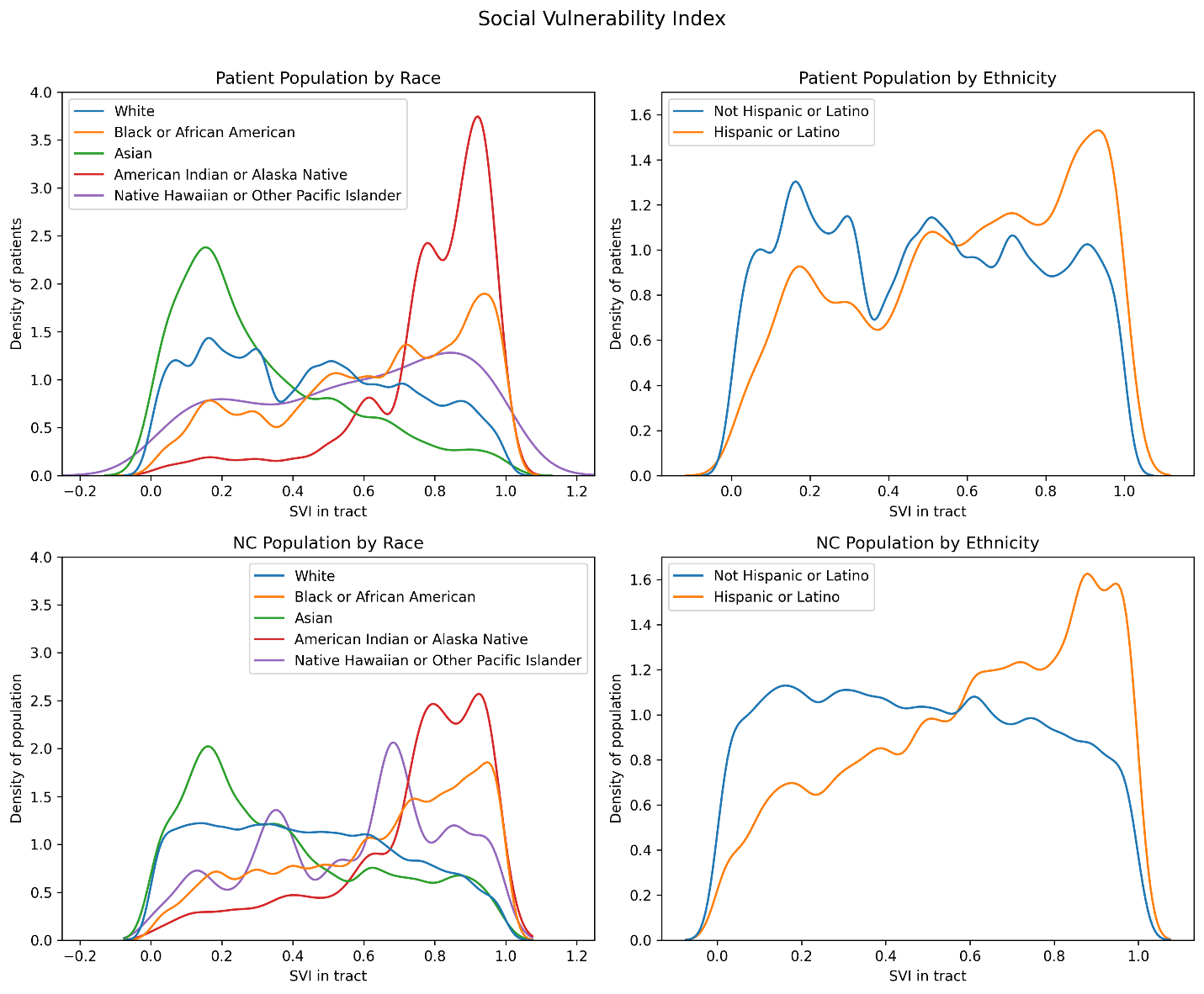


Figure S1. Kernel density estimation plots for Social Vulnerability Index. The Social Vulnerability Index is expressed as a ranking of tracts in NC, which is then scaled to a score range of 0-1. Higher scores indicate greater vulnerability to hazardous events. Curves indicate the density of the groups across tract scores. Curves are individually normalized by group size such that the area under each curve equals one, allowing for relative density comparisons. SVI = Social Vulnerability Index


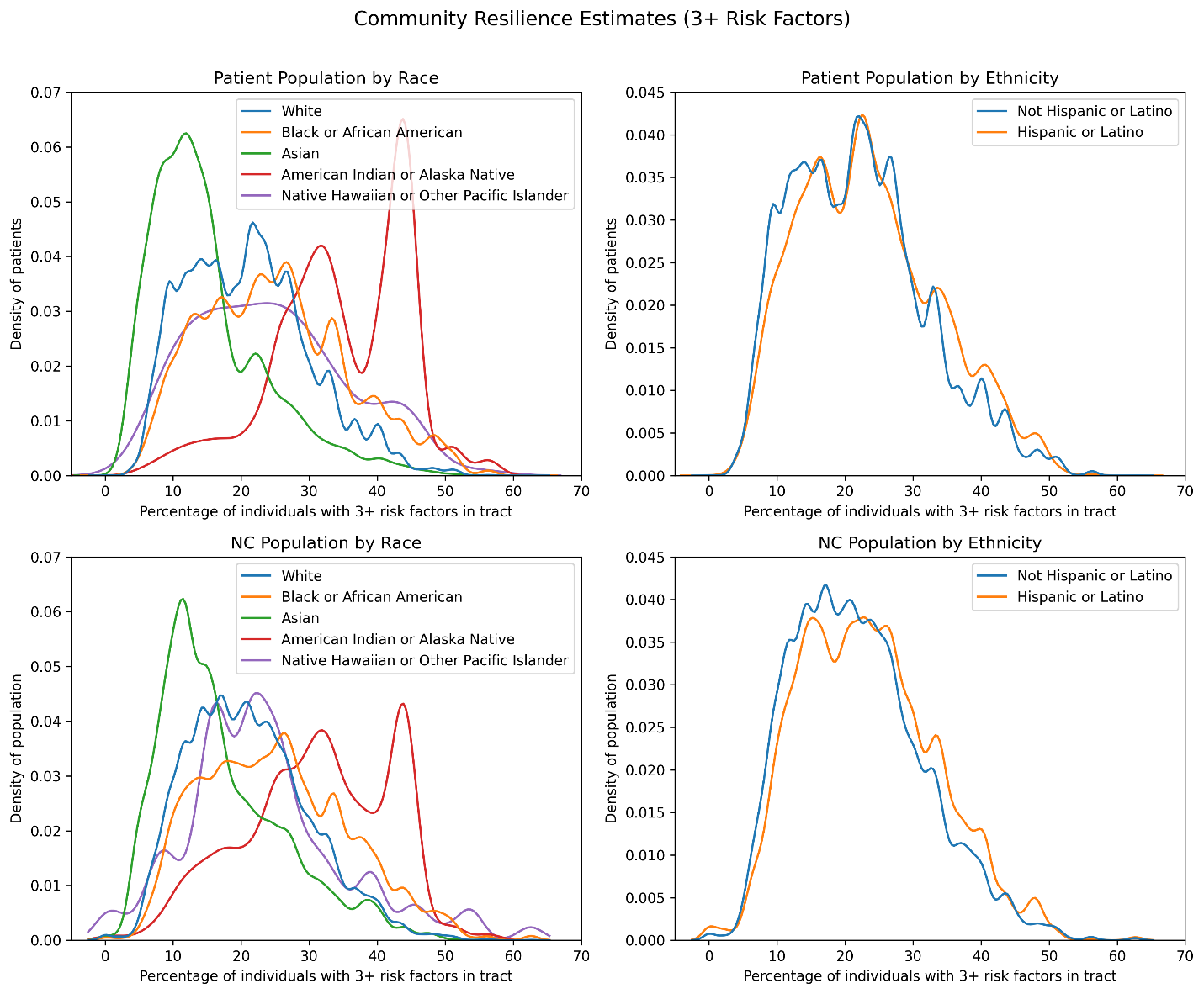


Figure S2. Kernel density estimation plots for Community Resilience Estimates. Community Resilience Estimates are expressed as the percentage of individuals in a tract with a given number of risk factors to being impacted by disasters. This figure illustrates percentage of individuals in a tract with three or more risk factors. Curves indicate the density of the groups across tract scores. Curves are individually normalized by group size such that the area under each curve equals one, allowing for relative density comparisons.


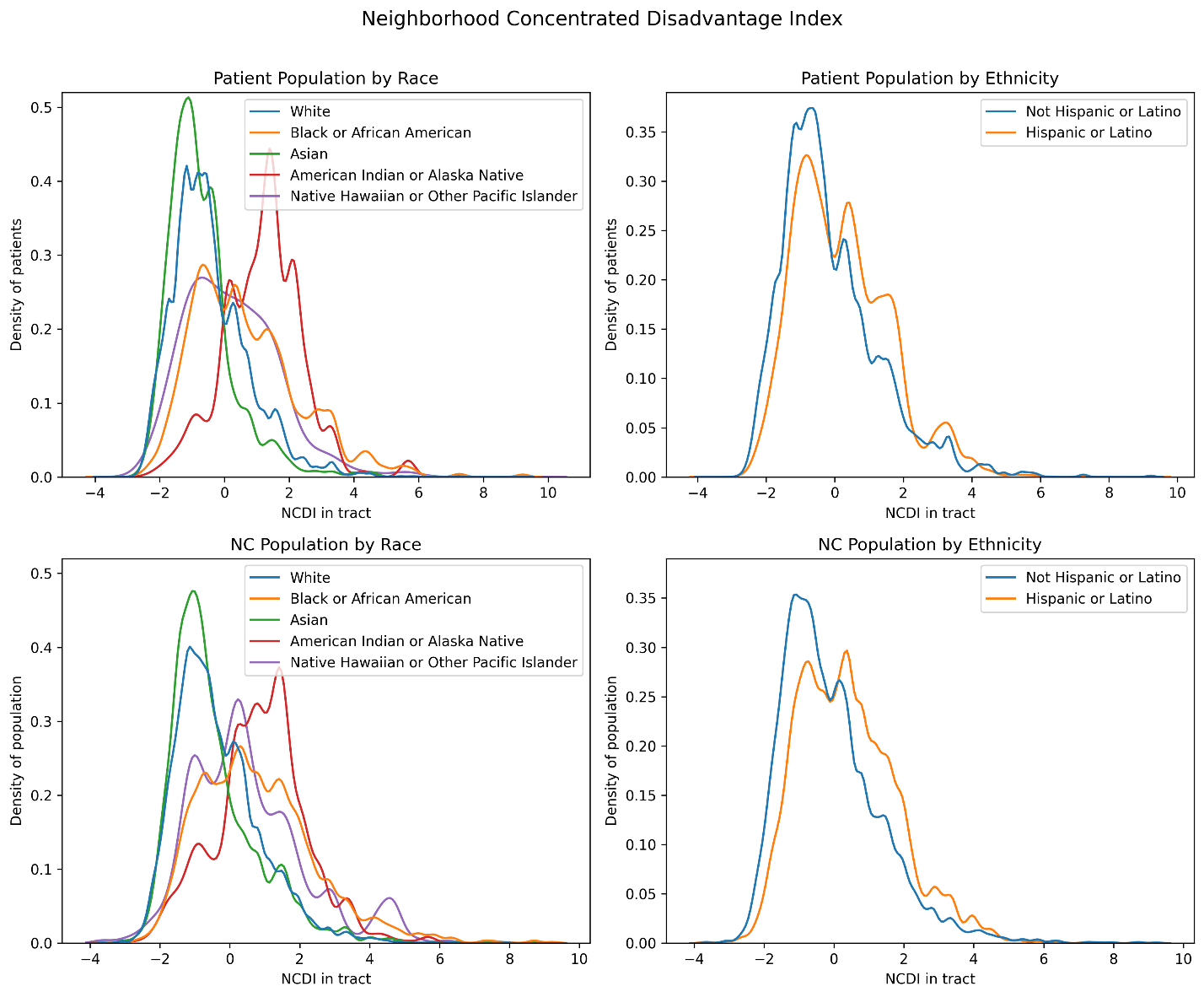


Figure S3. Kernel density estimation plots for Neighborhood Concentration Disadvantage Index. The Neighborhood Concentration Disadvantage Index is a weighted average of five variables from the ACS, with higher scores indicating greater disadvantage. Curves indicate the density of the groups across tract scores. Curves are individually normalized by group size such that the area under each curve equals one, allowing for relative density comparisons. NCDI = Neighborhood Concentration Disadvantage Index


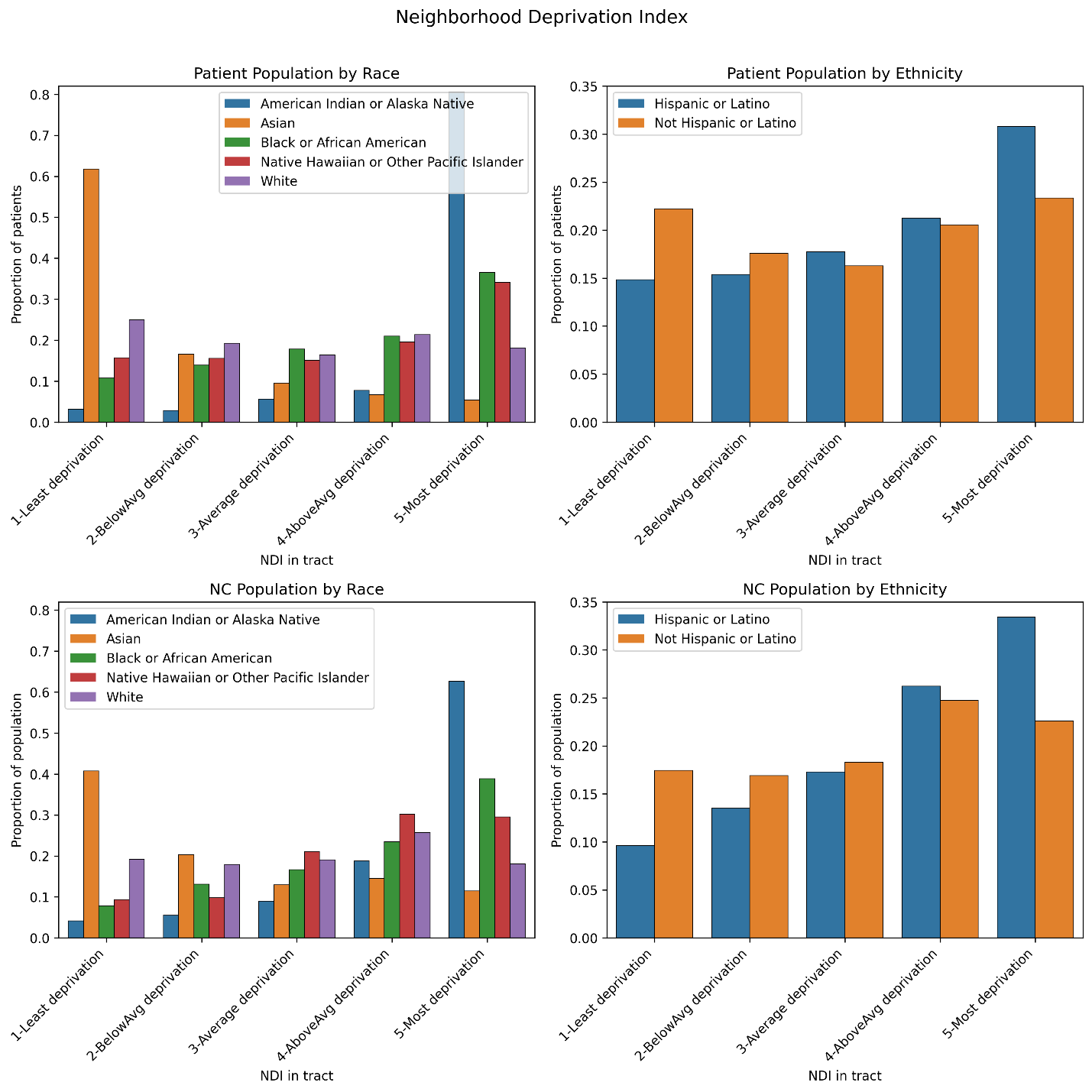


Figure S4. Bar plots for Neighborhood Deprivation Index. The Neighborhood Deprivation Index is expressed using five ordinal categories, with higher scores indicating greater deprivation. Bars indicate proportions by group, such that the bars for each group sum to one, allowing for relative comparisons. NDI = Neighborhood Deprivation Index


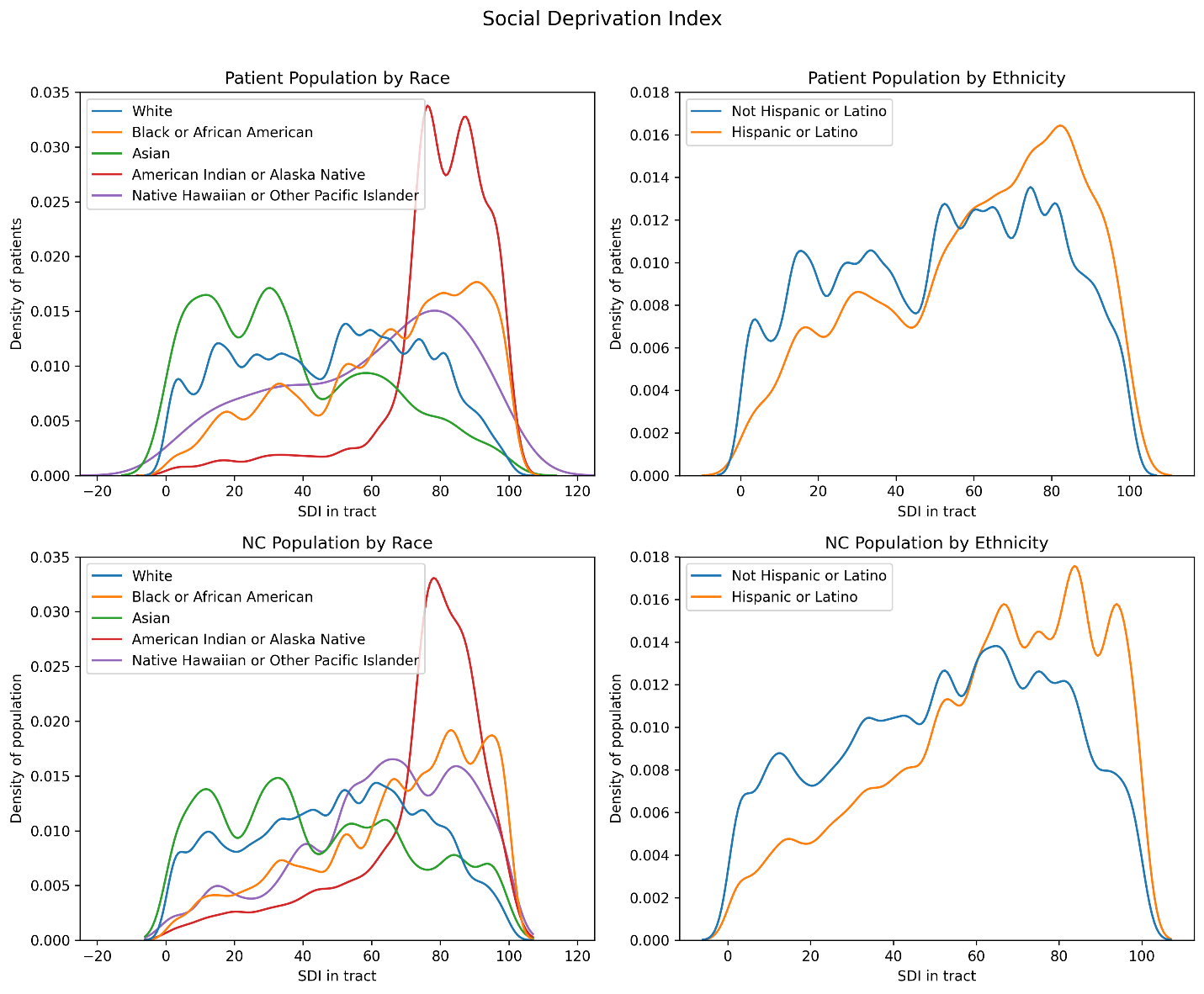


Figure S5. Kernel density estimation plots for Social Deprivation Index. The Social Deprivation Index is expressed as percentile scores, with higher scores indicating greater deprivation. Curves indicate the density of the groups across tract scores. Curves are individually normalized by group size such that the area under each curve equals one, allowing for relative density comparisons. SDI = Social Deprivation Index


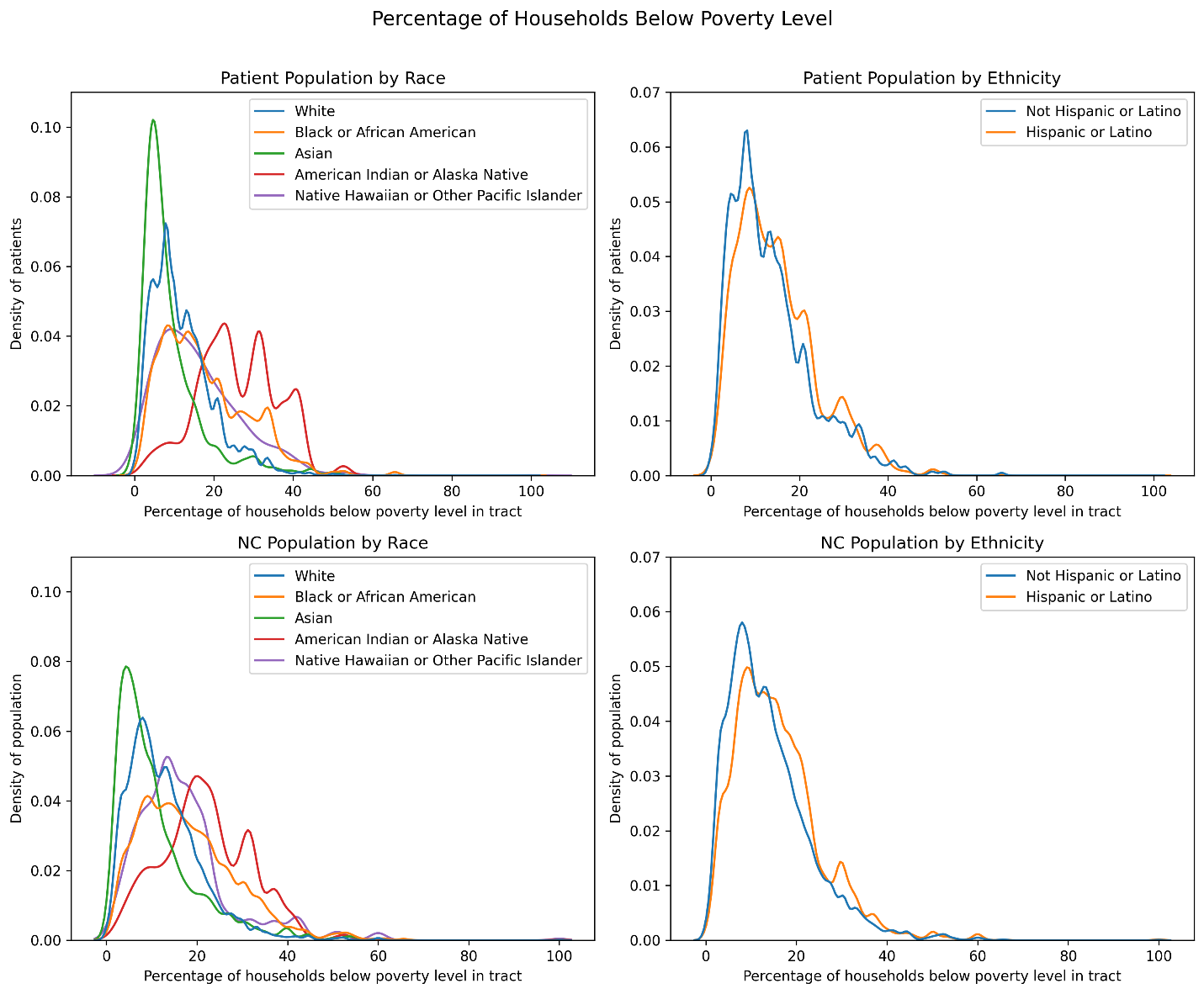


Figure S6. Kernel density estimation plots for percentage of households below the poverty level. Curves indicate the density of the groups across tract scores. Curves are individually normalized by group size such that the area under each curve equals one, allowing for relative density comparisons.
